# Dissociable Roles of the mPFC-to-VTA Pathway in the Control of Impulsive Action and Risk-Related Decision-Making in Roman High- and Low-Avoidance Rats

**DOI:** 10.1093/ijnp/pyae034

**Published:** 2024-08-19

**Authors:** Ginna Urueña-Méndez, Chloé Arrondeau, Florian Marchessaux, Raphaël Goutaudier, Nathalie Ginovart

**Affiliations:** Departments of Psychiatry and Basic Neurosciences, Faculty of Medicine, University of Geneva, Geneva, Switzerland; Departments of Psychiatry and Basic Neurosciences, Faculty of Medicine, University of Geneva, Geneva, Switzerland; Departments of Psychiatry and Basic Neurosciences, Faculty of Medicine, University of Geneva, Geneva, Switzerland; Departments of Psychiatry and Basic Neurosciences, Faculty of Medicine, University of Geneva, Geneva, Switzerland; Departments of Psychiatry and Basic Neurosciences, Faculty of Medicine, University of Geneva, Geneva, Switzerland

**Keywords:** Impulsive action, RDM, mPFC, VTA, [^18^F]-fluorodeoxyglucose

## Abstract

**Background:**

Impulsive action and risk-related decision-making (RDM) are associated with various psychiatric disorders, including drug abuse. Both behavioral traits have also been linked to reduced frontocortical activity and alterations in dopamine function in the ventral tegmental area (VTA). However, despite direct projections from the medial prefrontal cortex (mPFC) to the VTA, the specific role of the mPFC-to-VTA pathway in controlling impulsive action and RDM remains unexplored.

**Methods:**

We used positron emission tomography with [^18^F]-fluorodeoxyglucose to evaluate brain metabolic activity in Roman high- (RHA) and low-avoidance (RLA) rats, which exhibit innate differences in impulsive action and RDM. Notably, we used a viral-based double dissociation chemogenetic strategy to isolate, for the first time to our knowledge, the role of the mPFC-to-VTA pathway in controlling these behaviors. We selectively activated the mPFC-to-VTA pathway in RHA rats and inhibited it in RLA rats, assessing the effects on impulsive action and RDM in the rat gambling task.

**Results:**

Our results showed that RHA rats displayed higher impulsive action, less optimal decision-making, and lower cortical activity than RLA rats at baseline. Chemogenetic activation of the mPFC-to-VTA pathway reduced impulsive action in RHA rats, whereas chemogenetic inhibition had the opposite effect in RLA rats. However, these manipulations did not affect RDM. Thus, by specifically targeting the mPFC-to-VTA pathway in a phenotype-dependent way, we reverted innate patterns of impulsive action but not RDM.

**Conclusion:**

Our findings suggest a dissociable role of the mPFC-to-VTA pathway in impulsive action and RDM, highlighting its potential as a target for investigating impulsivity-related disorders.

**Significance Statement**

In this study, we showed that high-impulsive rats display reduced frontocortical glucose metabolism relative to low-impulsive rats. By altering the activity of the mPFC-to-VTA pathway, we were able to specifically revert an innate impulsivity pattern. Activation of the mPFC-to-VTA pathway in high-impulsive rats reduced impulsive action, while inhibition of the pathway in low-impulsive rats had the opposite effect. Interestingly, risk-related decision-making was unaffected, suggesting that both behavioral traits are controlled by different neural circuits. Our results indicate that the mPFC-to-VTA pathway is critically involved in the control of impulsive action, thus providing a promising avenue for studying disorders with impaired impulse control, such as substance use disorder.

## INTRODUCTION

The tendency to respond prematurely, that is, impulsive action, and to make decisions without considering potential risks, that is, risk-related decision-making (RDM), are normal personality traits. However, excessive impulsive action and RDM may contribute to psychiatric disorders such as drug abuse. Indeed, clinical research shows that individuals with psychostimulant abuse display heightened impulsive action (Voon et al., 2014) and RDM (Kjome et al., 2010) compared with healthy controls, and in animals, those behaviors respectively predict cocaine intake (Dalley et al., 2007; Arrondeau et al., 2023) and incubation of craving after drug discontinuation (Ferland and Winstanley, 2017). Given those findings, elucidating the neuronal circuits of impulsivity and RDM could help us understand the individual vulnerability to drug abuse.

The medial prefrontal cortex (mPFC) has been proposed as a key structure for regulating impulsive action and RDM. Neuroimaging studies show that individuals with mPFC lesions display higher self-reported impulsivity (McDonald et al., 2017) and RDM (Clark et al., 2008) than healthy controls. In healthy populations, reduced mPFC activity has been associated with heightened trait impulsivity (Boes et al., 2009; Neal and Gable, 2017), impulsive action (Neufang et al., 2016), and RDM (Lv et al., 2021). However, despite the association between mPFC activity and impulsive behaviors in humans, preclinical studies examining the causal role of the mPFC on impulsive action and RDM have yielded inconsistent results. For instance, in some studies, lesions or pharmacological inactivation of the infralimbic and prelimbic cortex (i.e., rodent homologs of the human mPFC) increased impulsive action (Chudasama et al., 2003; Murphy et al., 2012; Feja and Koch, 2014) and biased choices toward disadvantageous options (Paine et al., 2013; Zeeb et al., 2015; Orsini et al., 2018; van Holstein and Floresco, 2020). However, in other studies, lesions (Paine et al., 2013), pharmacological inhibition (Zeeb et al., 2015) or chemogenetic inhibition (van der Veen et al., 2021) of the mPFC did not affect impulsive action or RDM (Barrus et al., 2017). Given these discrepancies, further research is needed to clarify the contribution of the mPFC in modulating impulsive action and RDM.

The mPFC might modulate impulsive behaviors by exerting top-down control on subcortical structures (Dalley et al., 2011). Tracer studies (Carr and Sesack, 2000; Murugan et al., 2017; Souza et al., 2021) have shown that the mPFC sends direct projections to subcortical structures such as the dorsal striatum (DST), nucleus accumbens (NAc), and ventral tegmental area (VTA), which are critically involved in impulse control (Dalley and Robbins, 2017). For instance, studies in both humans and rodents have linked trait impulsivity (Buckholtz et al., 2010), impulsive action (Bellés et al., 2021; Urueña-Mendez et al., 2023), and RDM (Oswald et al., 2015) to heightened evoked dopamine (DA) release in the striatum. Additionally, in rodents, RDM has been associated with elevated VTA-DA neuronal firing. Thus, evidence suggests that the medial prefrontal cortex (mPFC) could affect impulsive action and RDM through pathway-specific projections. However, few studies have explored the role of specific mPFC projections on those behavioral traits, focusing primarily on impulsive action. For instance, in one study (de Kloet et al., 2021), chemogenetic inhibition of the mPFC-to-DST increased impulsive action, while in another study no significant changes in impulsive action occurred when inhibiting the same pathway (Nakayama et al., 2018). Critically, no studies, to our knowledge, have yet evaluated the role of the mPFC-to-VTA pathway in either impulsive action or RDM.

Interestingly, besides modulating striatal DA release (Hill et al., 2018), the mPFC can directly influence VTA-DA neuronal activity (Tan et al., 2014; Jo and Mizumori, 2016) and potentially impact impulsivity by controlling the activity of VTA-GABAergic neurons. Studies have suggested that reducing mPFC activity decreases the activity of putative GABAergic neurons, thereby potentiating DA neuron’s bursting firing (Tan et al., 2014; Jo and Mizumori, 2016). Thus, a reduced activity of the mPFC, as suggested in impulsive individuals, could underpin a reduced inhibition of dopaminergic neurons and consequently increased impulsivity. Building on this idea, here we assessed the effect of chemogenetic manipulations of the mPFC-to-VTA pathway in controlling impulsive action and RDM. We used the Roman high- (RHA) and low-avoidance (RLA) rat lines, which respectively display high- and low-impulsive behaviors (Bellés et al., 2023), to explore potential baseline differences in brain metabolism using in vivo positron emission tomography (PET) and the radiotracer [^18^F]-fluorodeoxyglucose ([^18^F]-FDG). We followed a double dissociation chemogenetic approach to assess the effects of targeted mPFC-to-VTA pathway activation or inhibition on impulsive action, RDM, and mPFC metabolic activity in RHA and RLA rats, respectively. We hypothesized that RHA rats have reduced mPFC-to-VTA activity and that activation of this pathway could therefore decrease their impulsive behavior. Likewise, we hypothesized that inhibition of this pathway in RLA rats might have the opposite effect.

## MATERIALS AND METHODS

### Subjects

Subjects were adult male RHA (n = 18) and RLA (n = 18) rats from our outbred colony at the University of Geneva. Rats were paired or trio housed in a temperature-controlled room (22°C ± 2°C) on a 12-hour-light/-dark cycle. Animals were maintained at 85% of their free-feeding weight during behavioral testing and received water ad libitum. All experiments were approved by the animal ethics committee of the Canton of Geneva and implemented according to the Swiss federal law on animal care.

### General Procedure

**Figure 1.**
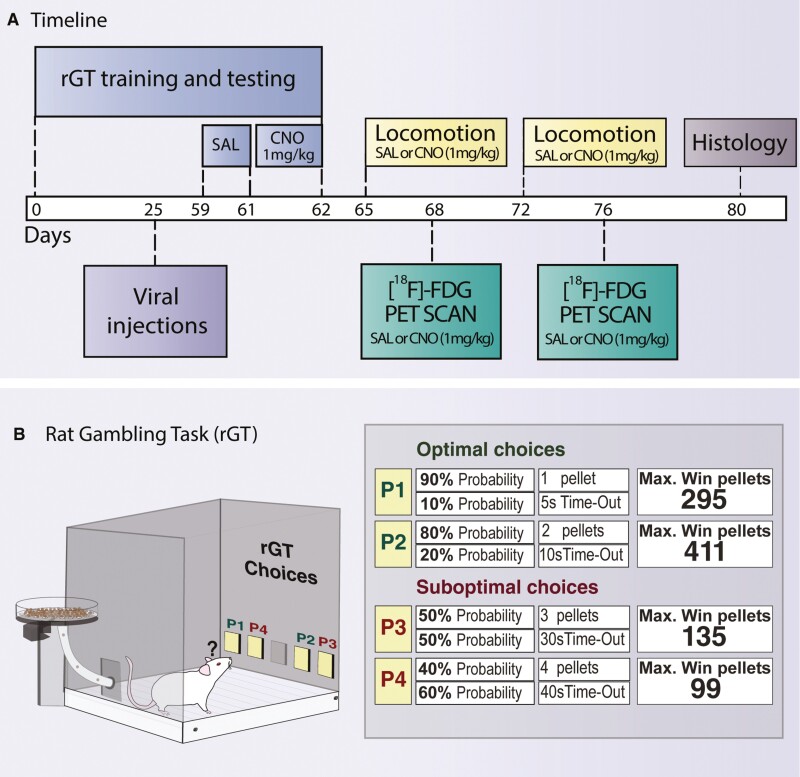
(A) Timeline of the study with annotated time points (in days). (B) Contingencies of the rat gambling task as described in Zeeb et al. (2009).

Figure 1A shows the timeline of the study. Rats were pretrained in the rat gambling task (rGT) for approximately 24 days. Then, they underwent stereotaxic surgery to specifically target the mPFC-to-VTA pathway. Rats received a retrogradely expressing Cre-recombinase virus in the VTA along with an anterograde Cre-dependent design receptor activated by a design drug (DREADD)-expressing virus in the mPFC. Specifically, RHA rats (n = 12) received an excitatory hM3Dq-DREADD, while RLA rats (n = 12) received an inhibitory hM4Di-DREADD. This approach allowed us to investigate whether line-specific targeting of the mPFC-to-VTA pathway modulates impulsive behaviors. Control animals expressing a Cre-dependent control protein instead of the DREADD were included for each rat line (n = 6). After surgical recovery, rats were trained in the rGT and tested until achieving stable performance (see rGT section). Rats then underwent testing sessions under saline vehicle and were subsequently tested under clozapine-N-oxide (CNO), the DREADD-activating drug. Three days after the last rGT session, rats performed 2 locomotion tests separated by a week, 1 under saline, and 1 under CNO, with the treatments counterbalanced. Finally, rats underwent PET scanning with [^18^F]-FDG to measure their brain metabolic activity under saline and CNO. Scans were conducted 1 week apart with counterbalanced drug administration.

### Drugs

CNO (HelloBio, Bristol, UK) was dissolved in 0.9% sterile saline at a concentration of 1 mg/mL and administered at 1 mg/kg 30 minutes before testing. The vehicle solution consisted of 0.9% sterile saline injected at 1 mL/kg.

### Stereotaxic Surgery and Viral Vectors

Details on surgical procedures are provided in the supplementary Methods. Rats were anesthetized and placed in a stereotaxic frame (RWD Life Science, Mainz, Germany). A craniotomy was performed at the target coordinates (VTA: AP = 3.7, ML = ±0.9, and DV = −7 mm. mPFC: AP = 12, ML = ±0.4, DV = −3.7 mm. AP was taken from interaural, and DV was taken from the dura). Viruses were delivered bilaterally (500 nL/site, at 3 nL/sec) using a nanoinjector (Nanoinject II, Drummond Scientific Company, PA, USA). In the VTA, all animals received an AAV-hSyn-EGFP-Cre-retrograde (8.8 × 10^12^ vg/mL, VVF, ETH, Switzerland). In the mPFC, viruses were infused as follows. For mPFC-to-VTA activation experiments, RHAs received AAV5-hSyn-DOI-hM3D(Gq)-mCherry (7 × 10¹² vg/mL, Addgene, plasmid number 44361). Conversely, for mPFC-to-VTA inhibition experiments, RLAs received AAV5-hSyn-DOI-hM4D(Gi)-mCherry (7 × 10¹² vg/mL, Addgene, plasmid number 44362). As a control, a subgroup of animals within each rat subline received AAV5-hSyn-DOI-mCherry (7.10¹² vg/mL, Addgene, plasmid number 50459). After injections, the scalp was sutured, and animals were allowed to recover for 1 week. Animals received pre- and postoperative analgesia and were fed ad libitum until resumed behavioral testing.

### The rGT

A detailed rGT protocol is presented in the supplementary Methods. In brief, rats were trained in daily 30-minute sessions as specified in Zeeb et al. (2009). Animals learned to nose-poke into 4 holes termed P1, P2, P3, and P4 (Figure 1B). The holes varied in the probability of receiving a specific number of food pellets or a time-out (TO) punishment of varying durations. On rewarded trials, rats received the specified number of pellets. On punished trials, TO was signaled by a light flashing at 0.5 Hz in the selected hole. P1 and P2 options were considered optimal as they resulted in more gains and less punishment in each session. Conversely, P3 and P4 were considered suboptimal as they resulted in fewer gains and more penalties in each session. Trials were separated by a 5-second intertrial interval; responses during this interval were considered premature. rGT training continued until choices stabilized (i.e., ≤25% variation in the rat’s choice score during 3 consecutive days). Next, animals were i.p. injected with saline 0.9% (1 mL/kg) 30 minutes before the session for 3 days. The average of those sessions served as a comparison baseline. The following day, rats received an i.p. injection of CNO (1 mg/kg) 30 minutes before testing.

A choice score [(%P1 + %P2) – (%P3 + %P4)] was calculated as an index of RDM (Zeeb and Winstanley, (2013)), with lower scores indicating less optimal decision-making. Impulsive action was measured as the percentage of premature responses (i.e., number of premature responses/total number of trials initiated × 100; Ferland and Winstanley, 2017).

### [^18^F]-FDG PET Scan

#### [^18^F]-FDG Image Acquisition and Reconstruction

Rats underwent 2 PET scans: 1 under saline and 1 under CNO. Scans were performed 1 week apart, and the treatments were administered in a counterbalanced order. Animals were fasted for 12 hours before each scan to minimize competition between dietary glucose and the [^18^F]-FDG radiotracer (Ribeiro et al., 2022). Rats then received an i.p. injection of CNO (1 mg/kg/mL) or an equal volume of saline vehicle. Next, rats were implanted with a polyurethane catheter in the tail vein and placed in polystyrene boxes for 30 minutes until receiving an i.v. bolus injection of 40 ± 5 MBq of [^18^F]-FDG (Department of Nuclear Medicine, Geneva University Hospitals). The doses injected did not significantly differ between rat lines or between saline and CNO conditions (RHA: SAL = 39.07 ± 4.52 MBq and CNO = 38.34 ± 6.38 MBq; RLA: SAL = 41.08 ± 4.65 MBq and CNO = 40.50 ± 4.11 MBq; line: F_(1,33)_ = 1.70, *P* = .20; time: F_(1,33)_ = 1.36, *P* = .25; line × time: F_(1,33)_ = 0.018, *P* = .89). After [^18^F]-FDG injection, rats were placed in standard home cages and kept warm with a red-light lamp to prevent brown tissue uptake of the radiotracer (Ribeiro et al., 2022). At 45 minutes uptake, anesthesia (2.5% isoflurane in oxygen) was initiated, and the rats (2 per scan) were positioned in the micro-PET scanner Triumph-II (TriFoil Imaging, Northridge, CA, USA) using a compatible custom-made stereotaxic-like frame. At 50 minutes post-radiotracer injection, animals were scanned for 20 minutes. Dynamic PET images were acquired in list mode and reconstructed into 4 time frames of 5 minutes each using the ordered subset expectation maximization algorithm with 20 iterations.

#### [^18^F]-FDG Analysis

Specific [^18^F]-FDG brain templates and regions of interest were created for each rat line using PMOD software (version 4, PMOD Technologies Ltd., Zurich, Switzerland) as described in the supplementary Methods. Individual PET scans were converted into standardized uptake values (SUV) according to the formula [R/(A/W)], where R is the radioactivity concentration (kBq/cc), A is the decay-corrected amount of injected radiotracer, and W is the weight of the rat in grams. Each PET scan was averaged to obtain corresponding PET summation images. Individual’s PET summation images were co-registered to the specific RHA or RLA brain template using mutual information-based rigid body registration, and the resulting transformation was applied to the corresponding PET dynamic scans. PET dynamic scans were then normalized using a whole-brain normalization factor (NF = average whole-brain uptake of all animals per group/individual whole-brain uptake), as previously described (Casado-Sainz et al., 2022). A specific template containing the following regions of interest—mPFC, midbrain, orbitofrontal cortex (OFC), cingulate cortex (Cg) DST, and ventral striatum (VST)—was then applied to the resulting normalized PET scans, and the normalized SUV (normSUV) were extracted per each region.

### Locomotor Activity

Locomotor testing occurred in 4 identical 48- × 48- × 40-cm transparent open-field boxes (ActiMot, TSE Systems, Bad Homburg, Germany). Tests were performed 1 week apart, once under saline and once under CNO (1 mg/kg). Treatments were administered in a counterbalanced order 30 minutes before testing, and the total traveled distance was recorded for 30 minutes.

### Tissue Preparation and Histology

Detailed information is provided in the supplementary Methods. Rats were transcardially perfused with 4% paraformaldehyde. Brains were extracted, frozen, and sliced into 40-μm coronal sections. Fluorescent images were acquired using a widefield fluorescence slide scanner microscope (Zeiss Axioscan Z1, Gottingen, Germany) and a confocal microscope (Zeiss LSM 800 airyscan).

### Statistical Analysis

Additional information is provided in the supplementary Methods. Statistical analyses were conducted with SPSS Statistics 26.0 (IBM) software. The normality of data distribution was verified using the Shapiro-Wilk statistic (*P < *.05), and for ANOVA analysis, non-normal data were LOG-10 transformed.

Baseline behavioral differences between rat lines (i.e., RHA vs RLA rats under saline) were analyzed with unpaired *t* tests or Mann-Whitney-U statistics, depending on the data distribution. Between-line differences in [^18^F]-FDG normSUV-values at baseline were analyzed using a repeated-measures ANOVA with rat line as between-subject factor and brain region as within-subject factor. Upon violation of data sphericity, the Greenhouse–Geisser correction was applied. The effect of mPFC-to-VTA pathway manipulation on behavioral measures was analyzed for each rat line using a repeated-measures ANOVA, with virus (i.e., DREADD, mCherry) as a between-subject factor and treatment (i.e., saline, CNO) as a within-subject factor. Additional analyses of the percentage of change under CNO were performed using unpaired *t* tests or Mann-Whitney-U statistics when appropriate. The effects of mPFC-to-VTA pathway manipulation on [^18^F]-FDG normSUV in the mPFC and midbrain regions were evaluated with a mixed factorial ANOVA with virus (i.e., DREADD, mCherry) as a between-subjects factor, and treatment (i.e., saline, CNO) and brain region as within-subjects factors. Due to a technical issue, the saline PET images for 1 RLA rat were lost; therefore, this animal was excluded from the PET analysis.

## RESULTS

### Baseline Differences in Impulsive Action, RDM, Locomotor Activity, and Brain [^18^F]-FDG Uptake in Roman Rats

**Figure 2.**
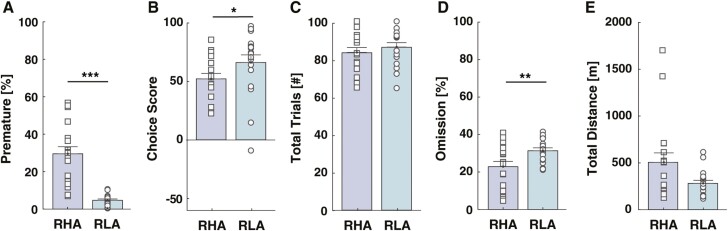
Baseline performances on the rat gambling task and locomotor activity in Roman rats. RHA rats (A) displayed a higher percentage of premature responses and (B) reached lower choice scores than RLA rats. (C) Both rat lines initiated a comparable number of trials. (D) RHA rats displayed a lower percentage of omissions. (E) No significant difference occurred in the total traveled distance during the locomotion test. However, RHA rats tended to travel greater distances than RLAs.

At baseline, RHA rats committed more premature responses (t_(36)_ = 6.4, df = 34, *P < *.0001, d = 2.15; Figure 2A; supplementary Figure 1) and reached lower choice scores than RLA rats (U_(36)_ = 94, *P* = .031, η^2^ = 0.13; Figure 2B; supplementary Figure 2), indicating that RHA rats display greater impulsive action and less optimal decision-making, respectively. While we observed no significant differences in each individual choice (supplementary Figures 3 and 4A), a further analysis of the percentage of optimal choices (%P1 + %P2) revealed a reduced preference for optimal options at baseline in RHA rats (U_(36)_ = 98, *P* = .044, η^2^ = 0.12; supplementary Figure 4B), thus explaining their lower choice scores. Although both rat lines initiated a comparable number of trials (t_(36)_ = −0.83, df = 34, *P* = .41, d = 0.04; Figure 2C), RHA rats committed fewer omissions (t_(36)_ = −2.66, df = 34, *P* = .01, d = 0.90; Figure 2D), suggesting that RHA rats have a higher motivation for the task. We also observed higher baseline locomotor activity in RHA than RLAs; however, this difference did not reach statistical significance (U_(36)_ = 107, *P* = .09, η^2^ = 0.08; Figure 2E).

**Figure 3.**
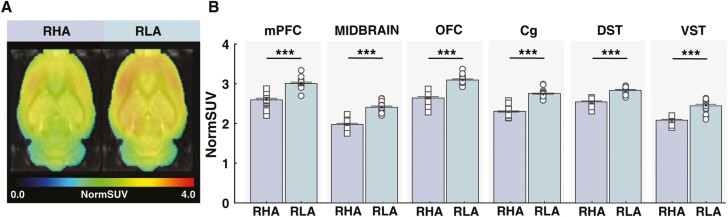
Baseline [^18^F]-FDG uptake in Roman rats. (A) Mean parametric maps of [^18^F]-FDG normalized standardized uptake values (normSUV) in RHA and RLA rats. NormSUV maps are projected on the MRI rat atlas (greyscale) and are shown in horizontal planes at the level of the mPFC. (B) RHA rats displayed lower [^18^F]-FDG uptake than RLA rats in the mPFC, midbrain, OFC, Cg, DST, and VST. Abbreviations: [^18^F]-FDG, [^18^F]fluorodeoxyglucose; Cg, cingulate cortex; DST, dorsal striatum; mPFC, medial prefrontal cortex; OFC, orbitofrontal cortex; VST, ventral striatum.

Mean parametric maps for baseline [^18^F]-FDG normSUV in RHA and RLA rats are presented in Figure 3A. As observed, RHA rats displayed lower [^18^F]-FDG normSUV compared with RLA rats. Such differences were observed along cortico-midbrain-striatal structures. There was a main effect of line (F_(1,33)_ = 268, *P* <.001, ηp^2^ = 0.99) and a line × region interaction (F_(3.5,115)_ = 2.94, *P < *.05, ηp^2^ = 0.08) on [^18^F]-FDG normSUV, which was significant for all evaluated brain regions (*P < *.001). These results indicate that RHA rats display lower metabolic brain activity than RLA rats.

### mPFC-to-VTA Pathway Modulation of Impulsive Action and RDM

**Figure 4.**
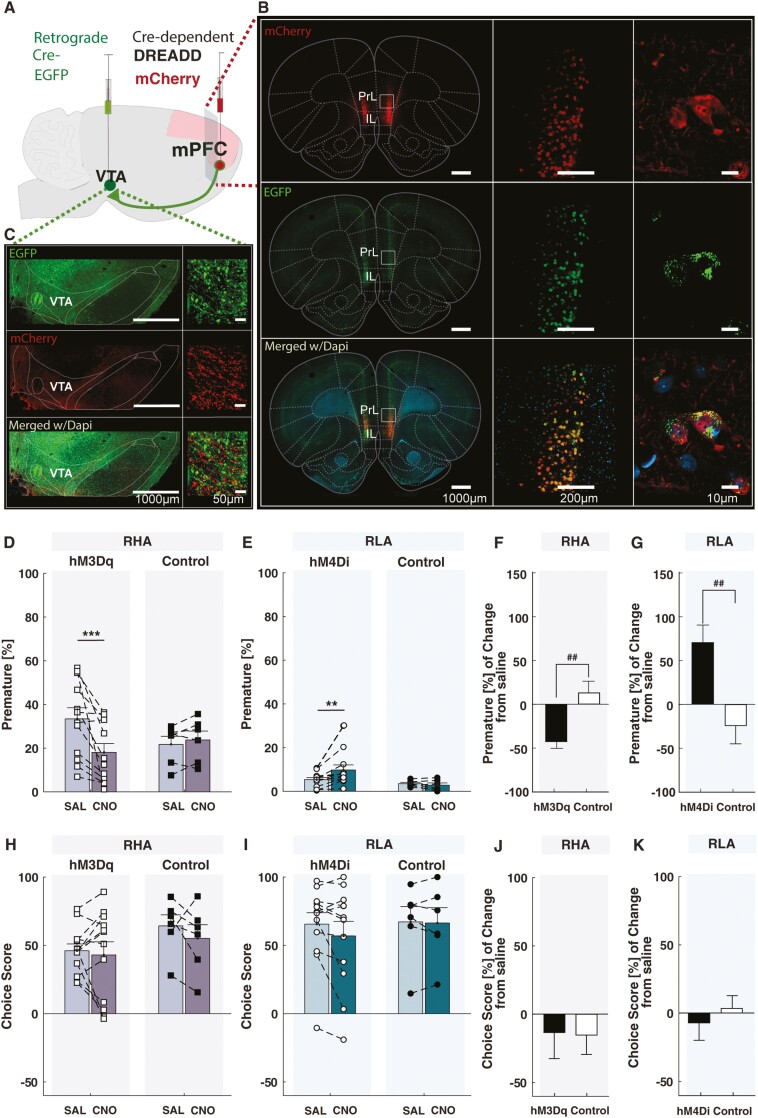
Chemogenetic manipulation of the medial prefrontal cortex to ventral tegmental area (mPFC-to-VTA) pathway on impulsive action and risk-related decision-making (RDM). (A) Viral injections. (B) Visualization of virally transduced mCherry fluorescence (red) in Cre-labelled neurons (green) within the medial prefrontal cortex (mPFC). (C) Double labeling of Cre-dependent retrograde (green) and mCherry-expressing terminals (red) within the VTA. (D) Chemogenetic activation of the mPFC-to-VTA pathway in RHA rats decreased premature responding. (E) Conversely, chemogenetic inhibition of the mPFC-to-VTA pathway in RLA rats increased premature responding. Similar results were observed when measuring the percentage of change in premature responses after clozapine-N-oxide (CNO) in (F) RHA and (G) RLA rats. Chemogenetic manipulation of the mPFC-to-VTA pathway did not alter the choice score in either (H) RHA or (I) RLA rats. Similar negative results were observed when measuring the percentage of change in choice score after CNO in (J) RHA and (K) RLA rats.

A representative image of the viral expressions in the mPFC-to-VTA is presented in Figure 4A–C. Manipulations of the mPFC-to-VTA pathway significantly affected premature responding (Figure 4D–G). In RHA rats, we observed a significant virus × treatment interaction (F_(1,16)_ = 7.25, *P* < .05, ηp^2^ = 0.31) but no main effects of virus (F_(1,16)_ = 0.22, *P* = .64, ηp^2^ = 0.01) or treatment (F_(1,16)_ = 4.28, *P* = .06, ηp^2^ = 0.2). Post-hoc comparisons revealed that CNO reduced premature responding in hM3Dq-expressing RHAs (*P* < .001) but not in controls (*P* = .80). An additional analysis of the percentage of change under CNO relative to saline further confirmed this result (hM3Dq-expressing vs control RHA rats: t_(18)_ = 4.01, df = 16, *P* < .01, d = 1.92), suggesting that the reduction in premature responding was specific to the mPFC-to-VTA pathway activation and not to nonspecific effects of CNO.

In RLA rats, there was also a significant virus × treatment interaction (F_(1,16)_ = 4.68, *P* < .05, ηp^2^ = 0.23) but not main effects of virus (F_(1,16)_ = 3.72, *P* = .07, ηp^2^ = 0.19) or treatment (F_(1,16)_ = 2.53, *P* = .13, ηp^2^ = 0.14). Post-hoc comparisons revealed that CNO increased premature responding in hM4Di-expressing RLAs (*P* < .01) but not in controls (*P* = .73). This result was further confirmed by an additional analysis of the percentage of change in premature responding under CNO relative to saline (hM4Di-expressing vs control RLA rats: U_(18)_ = 6, *P* < .01, η^2^ = 0.46), indicating that inhibition of the mPFC-to-VTA pathway, rather than nonspecific effects of CNO, increased premature responding. Altogether, our results suggest that activation of the mPFC-to-VTA pathway reduces impulsive action, while inhibition has the opposite effect.

When evaluating the effect of mPFC-to-VTA pathway manipulations on RDM (Figure 4H–K), we observed no virus × treatment interaction in either rat line (RHA: F_(1,16)_ = 0.28, *P* = .60, ηp^2^ = 0.02; RLA: F_(1,16)_ = 1.29, *P* = .27, ηp^2^ = 0.07). There were also no main effect of virus (RHA: F_(1,16)_ = 1.83, *P* = .19, ηp^2^ = 0.10; RLA: F_(1,16)_ = 0.13, *P* = .72, ηp^2^ = 0.01), nor a main effect of treatment in any rat line (RHA: F_(1,16)_ = 1.13, *P* = .30, ηp^2^ = 0.07; RLA: F_(1,16)_ = 1.84, *P* = .19, ηp^2^ = 0.10), suggesting that the activity of the mPFC-to-VTA pathway does not control RDM. Analysis of the percentage change in RDM under CNO, relative to saline, further revealed no significant differences between the DREADD-expressing and control animals in either rat line (RHA: t_(18)_ = 0.07, df = 16, *P* = .94, d = 0.03; RLA: t_(18)_ = 0.64, df = 16, *P* = .5, d = 0.36). Additionally, we observed no significant effects of chemogenetic manipulations when analyzed each individual options (supplementary Figure 5). Altogether, our results suggest that the mPFC-to-VTA pathway specifically modulates impulsive action but not RDM.

### mPFC-to-VTA Pathway Activity and Other Behavioral Measures

**Figure 5.**
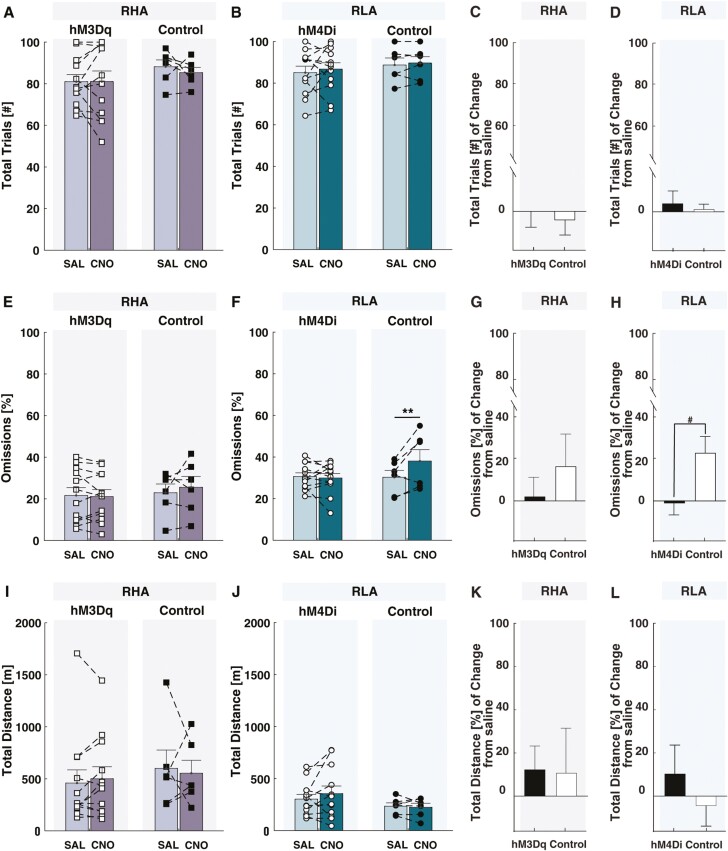
Chemogenetic manipulation of the medial prefrontal cortex to ventral tegmental area (mPFC-to-VTA) pathway on other behavioral measures. Chemogenetic manipulation of the mPFC-to-VTA pathway in RHA or RLA rats did not significantly alter (A–D) the total number of trials initiated and (E–H) the percentage of omissions in the rGT, or (I–L) the locomotor activity in an open field as measured by the total distance traveled.

Neither activation nor inhibition of the mPFC-to-VTA pathway affected the total number of trials initiated in the rGT (Figure 5A–D) in either rat line (virus × treatment in RHA: F_(1,16)_ = 0.31, *P* = .58, ηp^2^ = 0.02; in RLA: F_(1,16)_ = 0.04, *P* = .84, ηp^2^ = 0). There was also no main effect of virus (RHA: F_(1,16)_ = 0.91, *P* = .36, ηp^2^ = 0.02; RLA: F_(1,16)_ = 0.50, *P* = .49, ηp^2^ = 0.03) or effect of treatment (RHA: F_(1,16)_ = 0.31, *P* = .58, ηp^2^ = 0.02; RLA: F_(1,16)_ = 0.37, *P* = .55, ηp^2^ = 0.02). These findings were consistent with an analysis of the percentage of change under CNO, relative to saline, where no significant differences occurred between DREADD-expressing and control animals in either rat line (RHA rats: t_(18)_ = 0.35, df = 16, *P* = .72, d = 0.18; RLA rats: t_(18)_ = 0.31, df = 16, *P* = .76, d = 0.18).

Figure 5E to 5H shows the effect of mPFC-to-VTA pathway manipulations on the percentage of omissions. In RHA rats, we observed no virus × treatment interaction (F_(1,16)_ = 1.05, *P* = .32, ηp^2^ = 0.06), nor any main effect of virus (F_(1,16)_ = 0.25, *P* = .63, ηp^2^ = 0.02) or treatment (F_(1,16)_ = 0.46, *P* = .51, ηp^2^ = 0.03). There was also no significant change in omissions under CNO relative to saline between hM3Dq-expressing and control RHA rats (t_(18)_ = 0.84, df = 16, *P* = .41, d = 0.40). Conversely, in RLA rats, we observed a virus × treatment interaction (F_(1,16)_ = 7.43, *P* = .01, ηp^2^ = 0.32) and treatment effect (F_(1,16)_ = 5.10, *P* = .04, ηp^2^ = 0.4) on omissions. Post-hoc analyses revealed that omissions were higher only in RLA controls after CNO (*P* < .01). The percentage of change in omissions in CNO, relative to saline, was significantly increased only in RLA controls (t_(18)_ = 2.61, df = 16, *P* < .05, d = 0.13). This result might be secondary to a higher variability and lower n within this control group.

Finally, neither activation nor inhibition of the mPFC-to-VTA pathway affected locomotion activity (Figure 5I–L). We observed no virus × treatment interaction (RHA: F_(1,16)_ = 2.44, *P* = .63, ηp^2^ = 0.02; RLA: F_(1,16)_ = 0.22, *P* = .69, ηp^2^ = 0.01), nor any main effect of virus (RHA: F_(1,16)_ = 0.53, *P* = .37, ηp^2^ = 0.05; RLA: F_(1,16)_ = 0.66, *P* = .43, ηp^2^ = 0.04) or treatment (RHA: F_(1,16)_ = 0.05, *P* = .82, ηp^2^ = 0.03; RLA: F_(1,16)_ = 0.16, *P* = .69, ηp^2^ = 0.01) in either rat line. Analysis of the percentage of change in locomotion under CNO, relative to saline, showed no significant differences between DREADD-expressing and control animals in either rat line (RHA: t_(18)_ = 0.08, *P* = .94, d = 0.04; RLA: t_(18)_ = 0.86, df = 16, *P* = .4, d = 0.1). Taken together, our results further suggest that the effects of the mPFC-to-VTA manipulations on impulsive action are not related to changes in locomotor functions.

### Effects of mPFC-to-VTA Pathway Manipulations on [^18^F]-FDG Uptake

**Figure 6.**
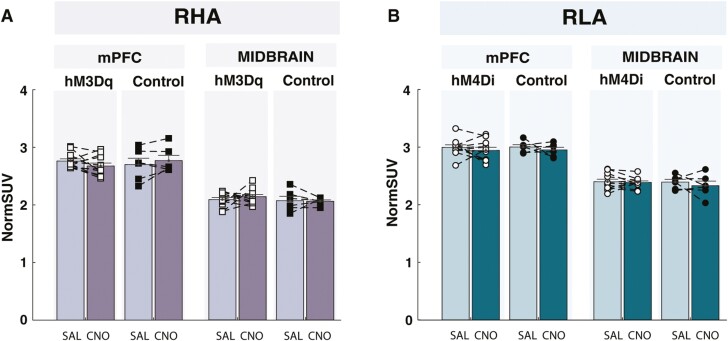
Chemogenetic manipulation of the medial prefrontal cortex to ventral tegmental area (mPFC-to-VTA) pathway on [^18^F]-FDG uptake. No alterations in [^18^F]-FDG uptake were detected in the medial prefrontal cortex (mPFC) or midbrain after chemogenetic manipulation of the mPFC-to-VTA pathway in either (A) RHA or (B) RLA rats.

Figure 6 presents [^18^F]-FDG NormSUV in the mPFC and midbrain after chemogenetic manipulations. Chemogenetic activation or inhibition of the mPFC-to-VTA pathway led to no virus × treatment × region interaction in RHA or RLA rats, respectively (RHA: F_(1,16)_ = 0.5 *P* = .49, ηp^2^ = 0.03; RLA: F_(1,15)_ = 0.33, *P* = .57, ηp^2^ = 0.02) or main effects of virus (RHA: F_(1,16)_ = 0.15, *P* = .71, ηp^2^ = 0.01; RLA: F_(1,15)_ = 0.13, *P* = .73, ηp^2^ = 0.01) or treatment (RHA: F_(1,16)_ = 0.03, *P* = .87, ηp^2^ = 0; RLA: F_(1,15)_ = 1.32, *P* = .27, ηp^2^ = 0.08). Altogether, our results suggest that chemogenetic manipulations of the mPFC-to-VTA pathway resulted in no detectable changes in [^18^F]-FDG uptake within the mPFC or midbrain.

## DISCUSSION

Our study offers new insights into the underlying mechanisms of impulse control by suggesting a direct contribution of the mPFC-to-VTA pathway in modulating impulsive action. Extending previous studies (Arrondeau et al., 2023; Bellés et al., 2023) showing greater impulsive action and less optimal decision-making in RHA than RLA rats, we observed reduced cortical [^18^F]-FDG uptake in the more impulsive phenotype. This result strengthens the view that reduced cortical function could be an underlying alteration to impulsive behaviors. Importantly, by specifically targeting the mPFC-to-VTA pathway in a phenotype-dependent way, we were able to revert innate patterns of impulsivity. Chemogenetic activation of the mPFC-to-VTA pathway in high-impulsive or RHA rats reduced premature responding, while chemogenetic inhibition of this pathway in low-impulsive or RLA rats had the opposite effect. Our findings indicated that the mPFC-to-VTA pathway selectively controls impulsive action, as neither chemogenetic manipulation altered RDM, further suggesting that these behavioral traits, while related, depend on separate neural circuits. Nevertheless, despite a significant behavioral effect on impulsive action, changes in brain [^18^F]-FDG uptake were undetectable. Taken together, our results support a regulatory role for the mPFC-to-VTA pathway on impulsive action, suggesting a potential target for understanding impulsivity-related disorders.

Our observation of lower brain [^18^F]-FDG uptake in RHA than RLA rats at baseline complements prior studies suggesting reduced glucose metabolism in the Cg and OFC of high-impulsive animals (Barbelivien et al., 2001). Moreover, our results extend previous studies showing that RHA rats exhibit reduced frontocortical volume (Río-Álamos et al., 2019) and decreased cortical glutamate receptors than RLA rats (Klein et al., 2014), supporting the idea that reduced frontocortical function might be linked to heightened impulsivity. Together with these reports, our observation of cortical hypometabolism in impulsive rats parallelled human studies, where individuals with high self-reported impulsivity (Matsuo et al., 2009; Neal and Gable, 2017) and RDM (Aydogan et al., 2021) exhibit reduced frontocortical activity and reduced cortical volume. Altogether, our results strengthen the view of frontocortical hypofunction as an alteration underlying impulsivity.

Although the mPFC exerts a top-down control on impulsivity-related structures (Dalley et al., 2011), no previous study to our knowledge explored the role of the mPFC-to-VTA pathway in the control of impulsive behaviors. By using a within-subject design and a chemogenetics strategy suited to the rats’ impulsive phenotype, our study shows for the first time, to our knowledge, that the mPFC-to-VTA has a specific modulatory role. Activating the mPFC-to-VTA pathway in RHA rats reduced their impulsive action, while inhibiting this pathway in RLA rats has the opposite effect. Notably, the absence of changes in overall locomotor activity after chemogenetic manipulations highlights the specificity of the mPFC-to-VTA pathway in the control of impulsive action. Our results are consistent with recent chemogenetic and optogenetic studies where nonspecific activation of the mPFC improved impulsive action (Warthen et al., 2016; Suri et al., 2023), while nonspecific inhibition impaired it (Luchicchi et al., 2016; Nakayama et al., 2018). Interestingly, our results extend those findings by indicating that the mPFC exerts a significant influence on impulsive action through its direct projections onto the VTA.

Impulsive action has been linked to dopaminergic function in the VTA (Hynes et al., 2021, 2024). High self-reported impulsivity in humans (Buckholtz et al., 2010) and heightened impulsive action in rodents (Bellés et al., 2021) have been associated with reduced availabilities of D2 autoreceptors in the VTA and increased evoked-DA release in the striatum. Moreover, in previous research, optogenetic stimulation of the VTA-DA projections to the NAc increased impulsive action (Flores-Dourojeanni et al., 2021), while chemogenetic inhibition—at least in males—reduced impulsivity (Hynes et al., 2021). Thus, it is possible that our manipulations of the mPFC-to-VTA pathway controlled impulsive action by affecting VTA-DA activity. Although the exact mechanism of mPFC modulation of VTA-DA activity remains unclear, studies have shown that mPFC can exert both excitatory and inhibitory effects on VTA-DA neurons (Tong et al., 1996). For instance, electrical stimulation of the mPFC increases VTA-DA firing (Lodge, 2011) and DA release in the NAc (Taber et al., 1995), but it can also inhibit a subset of VTA-DA neurons (Tong et al., 1996; Gao et al., 2007). This observation is consistent with pharmacological studies where mPFC inhibition increased VTA-DA firing (Patton et al., 2013; Jo and Mizumori, 2016). Interestingly, some studies proposed that mPFC activity inversely controls VTA-DA firing through the activation of VTA-GABA neurons (Tan et al., 2014). Considering that the mPFC directly innervates VTA-GABA neurons (Carr and Sesack, 2000; Sesack and Grace, 2010), such GABAergic control could be a potential circuit for the mPFC-to-VTA pathway regulation of impulsive action. This hypothesis aligns with a recent study suggesting that projections from cortical structures (i.e., the cingulate) to the VTA could inhibit DA neurons via activation of VTA-GABA interneurons (Song et al., 2024). However, future work is needed to confirm whether this cellular mechanism underpins the mPFC-to-VTA pathway effects on impulsive action.

Our results indicate that while the mPFC-to-VTA pathway modulates impulsive action, it does not appear to influence RDM. This selective control of the mPFC activity is consistent with previous studies using nonspecific mPFC-inactivation methods (Paine et al., 2013; Zeeb et al., 2015), suggesting that impulsive action and RDM may rely on distinct neuronal circuits. One possibility is that the mPFC-to-VTA pathway requires the convergent actions of other subcortical structures to control RDM. The rostromedial tegmental nucleus, known to inhibit VTA-DA neurons and control risk-reward seeking (Vento et al., 2017), is one potential candidate. Differential activity of the rostromedial tegmental nucleus between high- and low-impulsive rats could counteract any potential effect of the mPFC-to-VTA pathway manipulation on RDM. Alternatively, the mPFC might recruit different projections to influence RDM such as those innervating the NAc or the basolateral amygdala (Orsini et al., 2015; Truckenbrod et al., 2023). Further investigation targeting additional subcortical structures or mPFC projections would help determine the precise networks controlling RDM.

Chemogenetic manipulation of the mPFC-to-VTA pathway modulates impulsive action without inducing detectable changes in glucose metabolism. This result contrasts with previous research demonstrating detectable changes in brain glucose metabolism following chemogenetic manipulation of the nigrostriatal pathway (Casado-Sainz et al., 2022). One potential explanation for these differences is the relative density of projections within each specific pathway. While a precise quantification is currently lacking, it is reasonable to estimate that less than 20% of the mPFC neurons project to the VTA (Choi et al., 2024), compared with over 70% of the substantia nigra DA neurons projecting to the striatum (Chen et al., 2022). Thus, greater projection densities within the nigrostriatal pathway might have contributed to stronger or detectable brain metabolic changes. Altogether, our results suggest that while chemogenetic manipulation of the mPFC-to-VTA pathway influences impulsive action, its effect on brain glucose metabolism may lie below the detection threshold of the PET scanning.

Our study opens the possibility that alterations within the mPFC-to-VTA pathway in specific phenotypes play a role in impulsivity-related disorders such as drug abuse. Individuals with drug abuse disorders exhibit heightened impulsive action (Voon et al., 2014), frontocortical hypofunction (Volkow et al., 1992; Chen et al., 2007; Kim et al., 2009; Harlé et al., 2014), and disruptions in DA release (Martinez et al., 2007) compared with healthy controls. Interestingly, recent studies in rodents demonstrated that rats vulnerable to alcohol abuse (Gozzi et al., 2013) or compulsive cocaine seeking (Gozzi et al., 2011; Cannella et al., 2017; Jones et al., 2023) exhibit baseline alterations within cortical, striatal, and midbrain regions, consistent with those reported in impulsive or RHA rats. In line with these studies, our prior work indicates that high-impulsive or RHA rats are more vulnerable to the rewarding effects of cocaine (Dimiziani et al., 2019) and to cocaine-related alterations of DA function than low-impulsive or RLA rats (Urueña-Mendez et al., 2023). Thus, one interesting possibility is that the mPFC-to-VTA pathway activity not only controls impulsivity but lies at the intersection between impulsivity, frontocortical hypofunction, and individual vulnerability to drug abuse.

### Study Limitations and Future Research

We used [^18^F]-FDG PET as a non-invasive or indirect measure of large-scale brain activity. However, future studies might consider using additional molecular techniques, such as in vivo calcium imaging, which could provide a more sensitive measure of cortical activity in high- and low-impulsive rats during concurrent behavioral testing. Likewise, while we hypothesized that the mPFC-to-VTA pathway might affect impulsive action by acting on VTA-GABA neurons, confirming the VTA neuronal identity involved in this pathway would be necessary to support this hypothesis further. Additionally, although increased omissions in RLA controls might be due to the lower number of animals, off-target CNO effects on omissions have also been described in non-DREADD-expressing animals (Hynes et al., 2020). Thus, further studies might benefit from using additional techniques such as optogenetics to discard potential off-target CNO effects. Finally, a growing body of research suggests that the mechanisms underlying impulsivity might differ between males and females (Hynes et al., 2021, 2024; Orsini et al., 2022). For instance, as females display lower tonic DA levels, chemogenetic manipulations disrupting VTA-DA neuronal function could result in sex-mediated differences in overall DA levels and impulsivity (Hynes et al., 2021). Furthermore, it has been hypothesized (Hynes et al., 2024) that females may be more susceptible to the impulsivity-enhancing effects of DA-increasing manipulations, while males may exhibit greater reductions in impulsivity after DA-reducing manipulations. Additionally, the density of mPFC-to-VTA projections may differ between sexes, potentially amplifying differential CNO effects. Thus, extending our work by including female subjects is essential to determine whether sex influences the mPFC-to-VTA pathway’s control of impulsivity.

## CONCLUSION

Our study addresses a critical gap in evaluating the role of the mPFC-to-VTA pathway in regulating impulsivity. Using two rat lines with innate differences in impulsivity, we observed a hypocortical metabolism in the high-impulsive phenotype. Importantly, phenotype-specific chemogenetic manipulations of the mPFC-to-VTA pathway modulated impulsive action, but not RDM, indicating that these behavioral traits might be governed by distinct circuits. While more sensitive methods are required to determine the impact of the mPFC-to-VTA pathway manipulations on cortical activity, our study suggests a promising new target for investigating impulsivity-related disorders.

## Supplementary Material

pyae034_suppl_Supplementary_Materials

## Data Availability

The dataset used for the current study will be made available in Zenodo (https://zenodo.org) upon publication of the study.

## Notes

This study was funded by the Swiss National Science Foundation (SNSF; grant no.: 31003A_179373).
