# Supplementary material for: Dissociable Roles of the mPFC-to-VTA Pathway in the Control of Impulsive Action and Risk-Related Decision-Making in Roman High- and Low-Avoidance Rats
Source: Int J Neuropsychopharmacol. 2024 Aug 19;27(10):pyae034. doi: 10.1093/ijnp/pyae034 (PMC11450641; doi:10.1093/ijnp/pyae034)
Supplement: pyae034_suppl_Supplementary_Materials [file pyae034_suppl_supplementary_materials.docx]

**Supplementary methods**

Stereotaxic surgery and viral vectors

Rats were anesthetized with isoflurane in oxygen (3% during induction and 1% during maintenance) and placed on a heating pad in a stereotaxic frame (RWD Life Science, San Diego, USA). Their scalp skin was retracted to expose the skull and craniotomy was made at target coordinates in the VTA, [AP: 3.7, ML:±0.9 and DV:-7mm] and the mPFC [AP:12 ML: ±0.4 DV:-3.7 mm]. In both cases, the AP coordinate was taken from interaural, and the DV coordinate was taken from the dura. Viruses were delivered bilaterally (500nL per site) at a rate of 3nL per second, using a pulled glass capillary controlled by a nanoinjector (Nanoinject II, Drummond Scientific Company, Pennsylvania, USA). Before injecting, the capillary remained in the injection site for one minute. In the VTA, all animals received an AAV-hSyn-EGFP-Cre retrograde (8.8×1012 vg/mL, Viral Vector Facility, ETH, Switzerland). In the mPFC, viruses were infused as follows. For mPFC-to-VTA activation experiments, RHAs received AAV5-hSyn-DOI-hM3D(Gq)-mCherry (7×10¹²vg/mL, Addgene, plasmid number 44361). Conversely, for mPFC-to-VTA inhibition experiments, RLAs received AAV5-hSyn-DOI-hM4D(Gi)-mCherry (7×10¹²vg/mL, Addgene, plasmid number 44362). As a control, a subgroup of animals within each rat subline received AAV5-hSyn-DOI-mCherry (7.10¹² vg/mL, Addgene, plasmid number 50459). After injections, the capillary remained in place for 10 min before being slowly removed (~ 1 min). The scalp was sutured and animals were allowed to recover for one week. Animals received pre and post-operatory analgesia and were fed adlibitum until resumed behavioral testing.

Rat Gambling Task

The task was performed in eleven standard operant conditioning chambers (Med Associates Inc., St. Albans, VT, USA). Each chamber had a house light, a food tray, and five response holes (the central inactivated during testing). Each aperture was fitted with a cue light and infrared beams to detect nose-poke responses.

Rats were trained in daily 30 min sessions as specified in Zeeb (2009). Animals learned to nose-poke in the food tray to initiate each trial (pre-training). After 5s of inter-trial interval (ITI), one hole was illuminated, and the rat had 5s to nose-poke into that hole to obtain a food pellet reward (purified rodent tablets of 45 mg, Test Diet, Sandown Scientific, UK). Upon learning the nose-poke response, the rGT options were introduced over eight forced-choice (FC) sessions (four before stereotaxic surgeries and four after-surgery recovery). The rGT options were termed P1, P2, P3, and P4. They varied in the number of pellets delivered (1, 2, 3, or 4, respectively), the probability of pellet delivery (0.9, 0.8, 0.5, or 0.4, respectively), the time-out (TO) punishment probability (0.1, 0.2, 0.5, or 0.6, respectively) and the TO punishment duration (5s, 10s, 30s, or 40s, respectively). Each option was associated with specific holes counterbalanced between animals. On rewarded trials, rats received the specified number of pellets. There was no reward on punished trials, and TO punished was signaled with a light flashing at 0.5Hz in the selected hole. During each FC session, each option was individually presented in a pseudorandom order.

After the FC sessions, rats were trained in free-choice sessions of the rGT task, where the four options were simultaneously available. Free-choice training continued until the choice behavior stabilized (i.e., having ≤25% variation in the choice score during three consecutive days). Next, animals were i.p. injected with sterile saline 0.9% (1mL/kg) 30 min before the rGT testing for three days. The average of the three sessions served as a comparison baseline. The following day, rats received an i.p. injection of CNO (1mg/kg) 30 min before rGT testing.

*[^18^F]-FDG template generation*

[^18^F]-FDG brain templates for RHA and RLA rats were developed in PMOD software (version 4, PMOD Technologies Ltd., Zurich, Switzerland) according to a protocol previously described (Vállez Garcia et al., 2015). Individualized brain scans were obtained by cropping the double-rat dynamic PET scans. Next, individual PET scans were converted into SUV units according to the formula [R/(A/W)], where R is the radioactivity concentration (kBq/cc), A is the decay-corrected amount of injected radiotracer, and W is the weight of the rat in grams. Per each rat line, 10 individual scans were selected and one representative scan was used as a reference. Individual scans were normalized into the space of the reference and then were averaged into a single PET scan. This averaged scan was duplicated and flipped from left to right, and was then subsequently averaged with its flipped duplicated to create a symmetrical voxel-wise averaged template.

*Volumes of interest*

3D Volumetric atlas for each RHA and RLA [^18^F]-FDG template was constructed based on Schiffer’s magnetic resonance image (MRI) and volume of interest (VOI) atlas of the rat brain (Schiffer et al., 2006) as follows. First, the MRI atlas was coregistered to each rat template using mutual information-based rigid body registration. Next, the information outside the brain was masked in both the templates and the MRI. The masked MRI was then subjected to automatic elastic coregistration to each template dimension to obtain an adjusted MRI for each RHA and RLA template. The resulting transformations were independently applied to the VOI atlas so that the spatially transformed VOIs were specific for each rat line brain template.

A region of interest (ROI) was then defined on each adjusted MRI using the VOI atlas for RHA and RLA rats as a reference. The ROI template included the following brain regions: the medial prefrontal cortex (mPFC, 1mm x 1.2mm oval), the midbrain (1.7 mm x 0.6mm oval), the orbitofrontal cortex (OFC, 1.5mm x 0.7 mm oval ), the cingular cortex (Cg, 1.3mm x 1mm oval), the dorsal striatum (DST, 1.8mm circle) and the ventral striatum (VST, 1.2 mm circle). ROIs were placed on the central planes of each structure to minimize the partial volume effect.

**Tissue preparation and histology**

Rats were anesthetized using sodium pentobarbital (150 mg/kg, ip., at 200mg/mL) and transcardially perfused with 4% paraformaldehyde in 0.1M phosphate-buffered saline (PBS, pH=7.4). Brains were extracted and stored in 4% paraformaldehyde overnight at 4 °C and then transferred to a 30% sucrose solution at 4°C for 48-72h, before being snap-frozen in isopentane at -55°C. Brains were sliced into 40-μm coronal sections using a cryostat (CM3050, Leica Biosystem, Muttenz, Switzerland) and stored in a cryoprotectant solution at −20°C for further processing. Free floating sections were then washed in 0.1 M phosphate-buffered saline (PBS; 3 x 10 min). Following immersion in blocking solution (PBS, 0.3% Triton X-100, 10% BSA) at room temperature for 1h, sections were incubated with primary antibody against mCherry (sicgen AB0081-20, 1:500 dilution, overnight at 4°C). After washing (PBS, 3 x 10 min), sections were treated with secondary antibody (Abcam ab150135, 2 h, room temperature), and then counterstained with Hoechst 33342 (1:1000; 15 min, room temperature). Stained sections were washed (PBS, 3 x 10 min) and mounted on slides covered with antifade mounting medium (Fluorsave, Merck, Darmstadt, Germany). Stained sections were imaged at 10X using a widefield fluorescence slide scanner microscope (Zeiss Axioscan Z1, Gottingen, Germany). Magnification images were obtained at 20X and 63X using a confocal microscope (Zeiss LSM 800 airyscan).

**Statistical analysis**

Statistical analyses were conducted with SPSS Statistics 26.0 (IBM) software. The normality of data distribution was verified using the Shapiro-Wilk statistic (p<0.05), and for ANOVA analysis, non-normal data were LOG-10 transformed. Upon violation of data sphericity, the Greenhouse–Geisser correction was applied.

**Longitudinal analysis of rGT variables**

rGT variables, including premature responding, choice score, and individual (P1, P2, P3 and P4) choices were also analyzed along the rGT training (i.e., when the rats were exposed to the four options simultaneously). As the number sessions before reaching stability varied between the animals, to extract average curves per group we used the following approach. To standardize the analysis, data were aligned to the last session (L) before the saline administration and backtracked (L-1, L-2, etc) up to L-25 sessions. This alignment ensures that equivalent time points were compared across animals, regardless of their individual training timeline. We have also included the three saline sessions (S1, S2, S3) to perform comparisons between the final rGT training sessions and the saline administration period. We used a repeated measures ANOVA per each rat line, with line (RHA or RLA) as between subject’s factor and session (L-15 to S3) as within-subjects factor. This approach allowed us to evaluate a longitudinal performance patterns while accounting for differences in the number of sessions performed before reaching stability.

**Individual choices in RHA vs RLA rats at baseline**

In individual choices between RHA and RLA rats at baseline (i.e., the average of the three saline sessions) were evaluated using a two-way repeated measures ANOVA with line (RHA or RLA) as between subjects factor and individual choice (P1, P2, P3 and P4) as a within-subjects factor. Additionally, further differences in the percentage of optimal choices between RHA vs RLA rats were evaluated using Mann-Whitney-U statistics.

**CNO effects on individual choices**

The effect of mPFC-to-VTA pathway manipulation on each individual choice was analyzed for each rat line using a repeated measures ANOVA, with virus (i.e., DREADD, mCherry) as a between-subjects factor and treatment (i.e., saline, CNO) and choice (P1, P2, P3 and P4) as a within-subjects factors.

**Supplementary Results**

**Longitudinal performance of RHA and RLA on impulsive action and RDM**

A longitudinal analysis of the premature responses **(Fig. S1)** revealed a main effect of line (F_(1,34)_=50.52, p<0.0001, ηp^2^= 0.59) and session (F_(8,269)_= 2.88, p=0.004, ηp^2^=0.08), but no session x line interaction (F_(8,269)_= 0.81, p=0.5, ηp^2^=0.023). This result indicates that RHA rats were overall more impulsive than RLAs throughout the rGT training and testing.


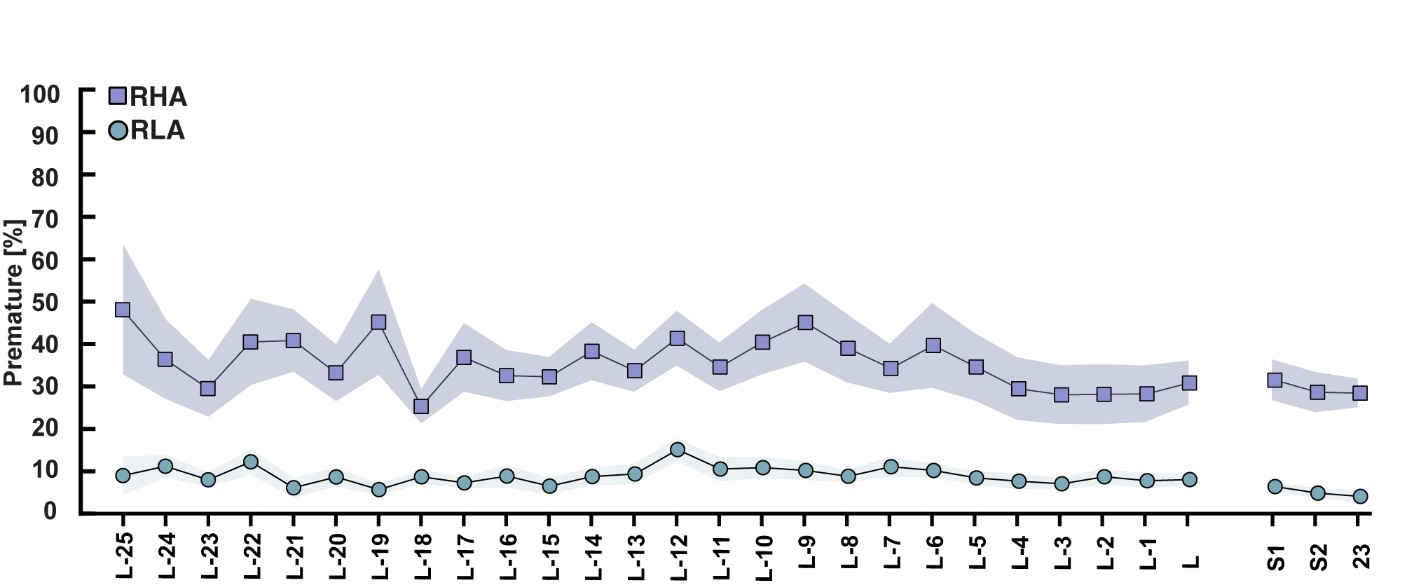


**Figure Supplementary 1**. Longitudinal analysis of premature responses during the rGT training and saline sessions. Data appear as mean ± SEM.

When analyzing the choice score **(Fig. S2),** we observed a significant effect of line (F_(1,34)=_ 4.1, p=0.05, ηp^2^= 0.11), but no effect of session (F_(5,168)=_ 0.81, p=0.5, ηp^2^= 0.056) and no session x line interaction (F_(5,168)=_ 0.81, p=0.5, ηp^2^= 0.02), suggesting that RHA rats have a less optimal decision-making independent of the training session.


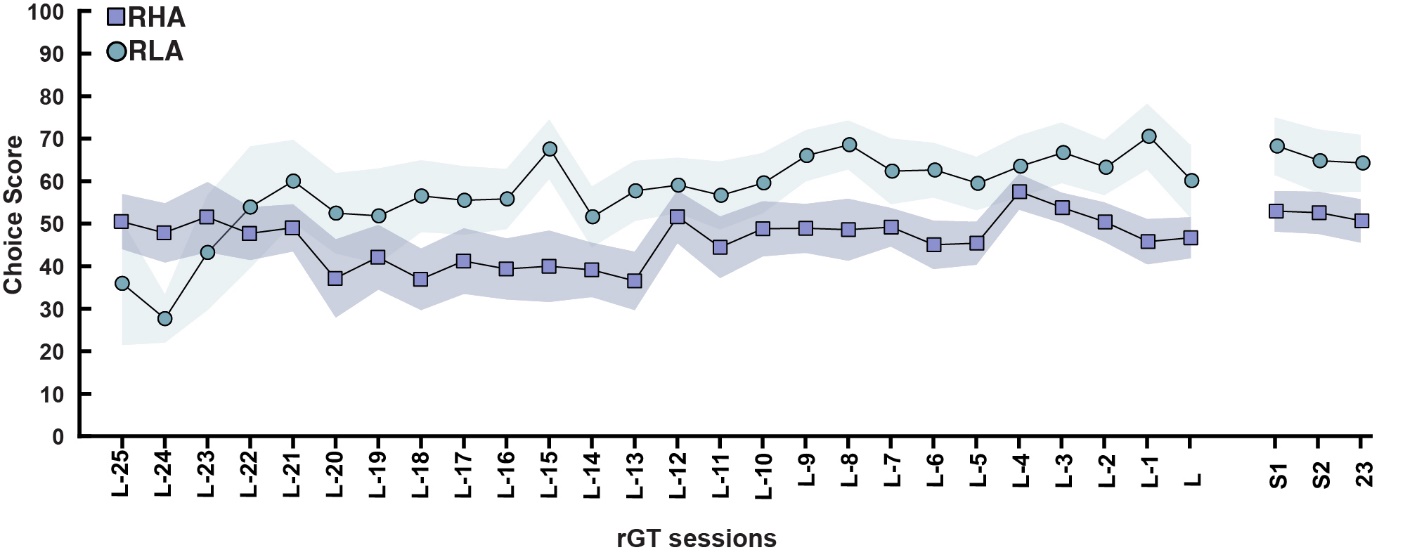


**Figure Supplementary 2**. Longitudinal analysis of the choice score durring the rGT training and saline sessions. Data appear as mean ± SEM.

Interestingly, analysis of individual choice options **(Fig. S3)** revealed no main effects of line (P1: F_(1,34)=_0.76, p=0.39, ηp^2^=0.02; P2: F _(1,34)=_2.82, p=0.10, ηp^2^=0.07; P3: F _(1,34)=_0.60, p=0.44, ηp^2^=0.01; P4: F _(1,34)=_4.02, p=0.06, ηp^2^=0.11). There was also no effect of session (P1: F_(6,202)_=13.8, p=0.22, ηp^2^=0.03; P2: F_(4,140)_=2.11, p=0.08, ηp^2^=0.06; P3: F_(9,297)_=1.42, p=0.18, ηp^2^=0.04; P4: F_(8,284)_=1.85, p=0.06, ηp^2^=0.05) nor any session x line interaction (P1: F_(6,202)_=1.34, p=0.24, ηp^2^=0.04; P2: F_(4,140)_=1.35, p=0.25, ηp^2^=0.04; P3: F_(9,297)_=0.68, p=0.71, ηp^2^=0.02; P4: F_(8,284)_=1.47, p=0.16, ηp^2^=0.04). These results suggest that the overall differences in decision-making between the lines is not be driven by the preference for a specific option but rather by a broader pattern of less optimal decision-making in RHA rats.


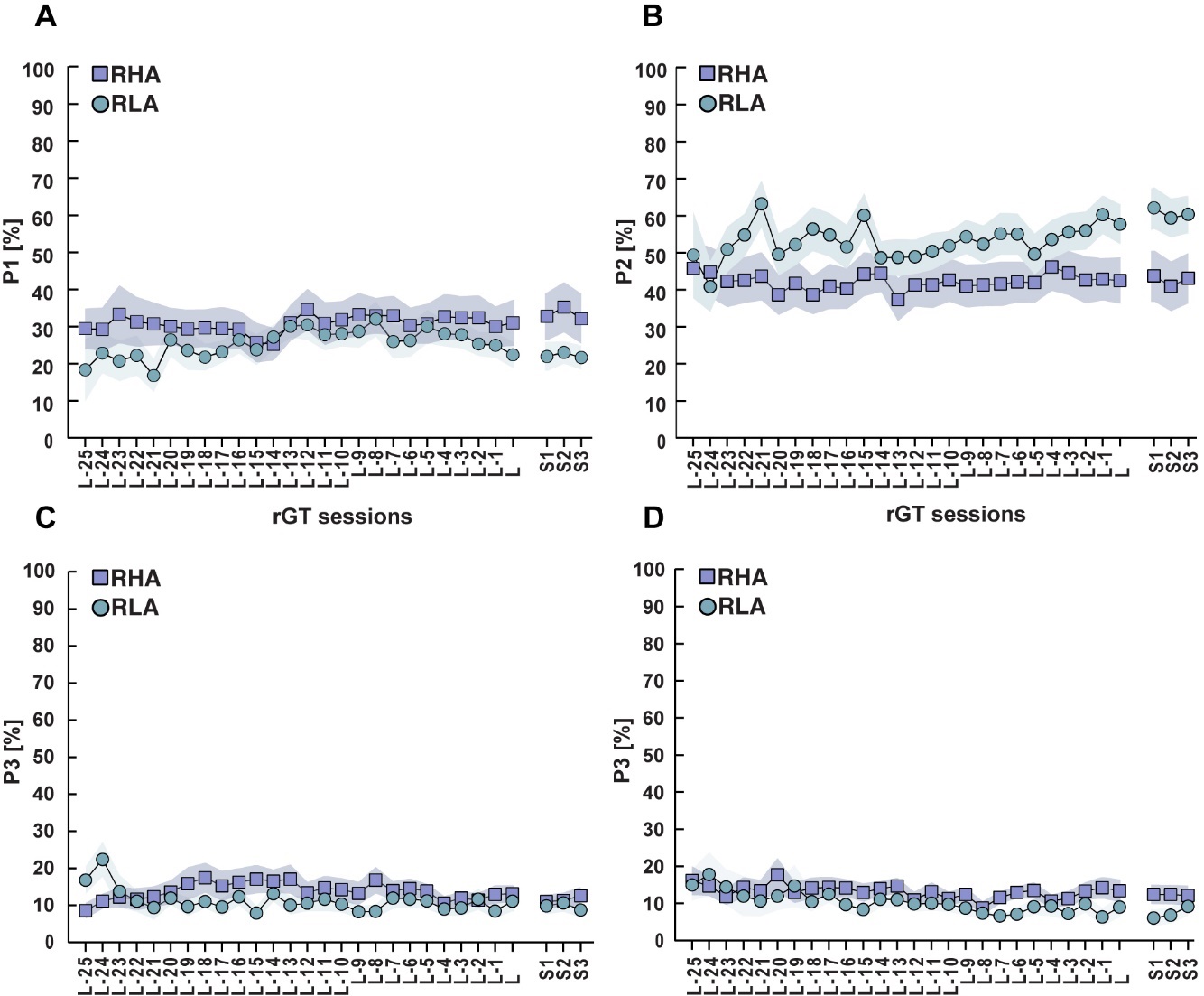


**Figure Supplementary 3**. Longitudinal analysis of each individual choice score durring the rGT training and saline sessions. A) %P1, B) %P2, C) %P3 and D) %P4. Data appear as mean ± SEM.


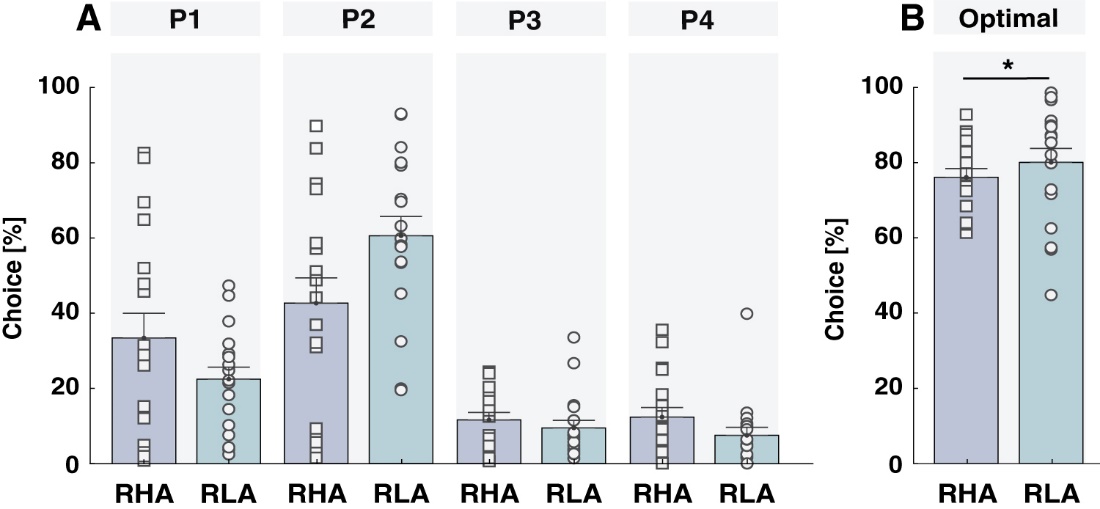
Further analysis of the individual choice options at baseline (Fig. S4. A) revealed that although both rat lines choose P1 and P2 more than P3 and P4, RHA rats tended to display less preference for the optimal options (choice x line interaction F_(1,47.12)_=3.45, p=0.056, ηp^2^=0.09). This was confirmed by a comparison of the percentage of optimal choices (Fig. S4. B), which indicated that RHA rats have a lower preference for P1 and P2 than RLA rats (U_(36)_=98, p=0.044, η^2^=0.12).

Figure Supplementary 4. A. individual options at baseline, B. Percentage of optimal choices at baseline. Data appear as mean ± SEM. Significantly different at *p<0.05, in RHA-vs-RLA rats.

**CNO effects on individual rGT options**

We then compared the CNO effect on the individual rGT options between DREADD-expressing and controls in each rat line (**Fig. S5. A-B)** we observed a significant effect of choice (RHA: F_(1,32.27)_=6.93, p=0.005, ηp^2^=0.3; RLA: F_(1,30.87)_=19.7, p<0.0001, ηp^2^=0.55), with an overall preference for optimal options in both lines. However, there was no main effect of treatment (RHA: F_(1,32.27)_=1.23, p=0.28, ηp^2^=0.07; RLA: F_(1,30.87)_=1.10 , p=0.3, ηp^2^=0.06), or virus (RHA: F_(1,16)_=3.12, p=0.1, ηp^2^=0.16; RLA: F_(1,16)_=0.001, p=0.97, ηp^2^=0.0), nor any a choice x treatment x virus interaction (in RHA: F_(1,32.27)_=2.33, p=0.11, ηp^2^=0.12, in RLA: F_(1,30.87)_=2.3 , p=0.12, ηp^2^=0.12). These results indicated that neither activation nor inhibition of the mPFC-to-VTA pathway significantly affected the choice of individual rGT options.


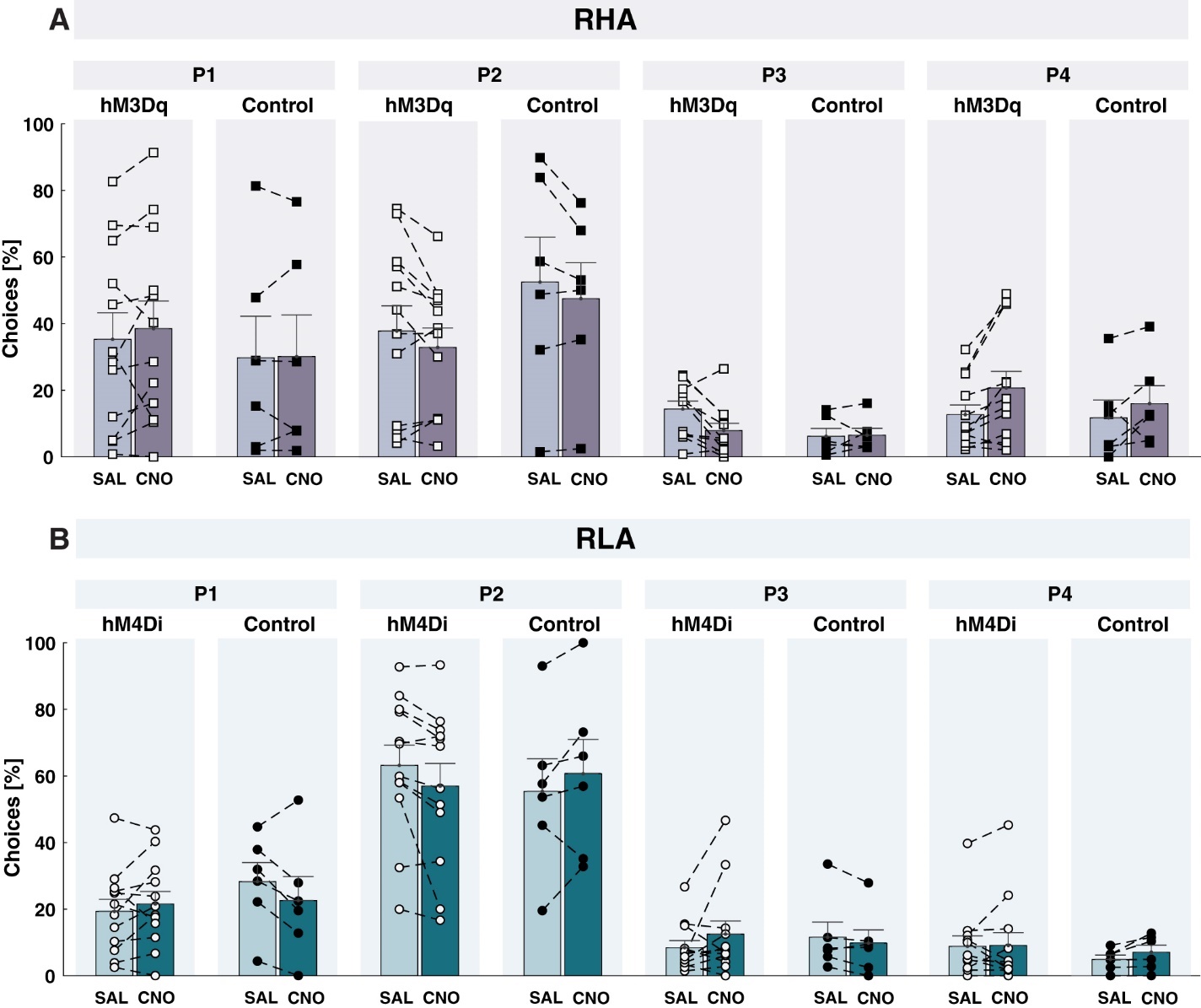


**Figure Supplementary 5.** Chemogenetic manipulation of the mPFC-to-VTA pathway on individual rGT options in **A)** RHA and **B)** RLA rats.

References

Schiffer WK, Mirrione MM, Biegon A, Alexoff DL, Patel V, Dewey SL (2006) Serial microPET measures of the metabolic reaction to a microdialysis probe implant. J Neurosci Methods 155:272–284.

Vállez Garcia D, Casteels C, Schwarz AJ, Dierckx RAJO, Koole M, Doorduin J (2015) A standardized method for the construction of tracer specific PET and SPECT rat brain templates: Validation and implementation of a toolbox. PLoS One 10:1–21.

Zeeb FD, Robbins TW, Winstanley CA (2009) Serotonergic and dopaminergic modulation of gambling behavior as assessed using a novel rat gambling task. Neuropsychopharmacology 34:2329–2343.
